# Case report: Bipolar disorder in 48,XXYY syndrome

**DOI:** 10.3389/fpsyt.2022.1080698

**Published:** 2023-01-11

**Authors:** Nur Atikah Razali, Tuti Iryani Mohd Daud, Luke Sy-Cherng Woon, Suriati Mohamed Saini, Noor Azimah Muhammad, Shalisah Sharip

**Affiliations:** ^1^Department of Psychiatry, Faculty of Medicine, Universiti Kebangsaan Malaysia Medical Centre, Kuala Lumpur, Malaysia; ^2^Department of Family Medicine, Faculty of Medicine, Universiti Kebangsaan Malaysia Medical Centre, Kuala Lumpur, Malaysia

**Keywords:** case report, bipolar mood disorder, metabolic syndrome, aggression, 48, XXYY syndrome

## Abstract

48,XXYY syndrome is a rare condition. The presentations of impulsive and aggressive behavior have been reported in several case reports among patients with 48,XXYY syndrome. The management of the psychological impact and neuropsychiatric sequela of this condition is a significant issue faced by families, carers, and healthcare professionals. We report a patient, 21-year-old Malay male, with underlying 48,XXYY syndrome with attention deficit hyperactivity disorder (ADHD) and intellectual disability, diagnosed later in adulthood with a bipolar mood disorder and benefited after being started on lithium. We describe the key clinical features and diagnostic workouts that allowed the arrival of the correct psychiatric diagnosis. Challenges in psychopharmacotherapy, including the risks of metabolic syndrome and deep vein thrombosis associated with 48,XXYY syndrome, are also considered. We suggest that for patients with 48,XXYY syndrome, routine psychological screening for mood symptoms such as mania and depression should be done by healthcare professionals with early involvement of psychiatrist in the multidisciplinary team due to the challenges in the management of these patients.

## 1. Introduction

48,XXYY is one of the most under-studied and rare types of sex chromosome aneuploidies (SCAs). In male births, 48,XXYY incidence occurs with an estimated prevalence of 1 in 18,000–40,000 (1). The SCAs can influence the neurodevelopment of an individual and are associated with impairment in executive function, verbal skills, working memory, sustained attention, mental flexibility, and inhibition by altering the basic differentiation process of the neurons, encoding proteins, and synaptic transmission (2). A study also shows that the genes in the sex chromosome may influence psychiatric disease (2). Currently, our understanding of the genetic mechanism behind the influence of SCAs, including 48,XXYY on the development of bipolar disorder is still very limited (2). However, there was a related explanation of the dynamic changes of x-linked escaping genes for the unmatched phenotype with aneuploidies. A possible mechanism for the development of psychiatric disorders between the patients with SCAs (Klinefelter syndrome XXY and Triple X syndrome XXX) and the general population of female with psychiatric disorders including bipolar disorder is shown by a study of human lymphoblastoid cells (3). This study found a significant over-expressed *XIST* gene, a master in control of X chromosome inactivation (XCI), in the lymphoblastoid cells of female patients with either bipolar disorder or major depression.

It has been suggested that aggression and delinquent behavior were more likely to be exhibited by patients with 48,XXYY, in comparison to other chromosomal aneuploidies such as 48,XXXY and 49,XXXXY (4). Mood, behavior, memory, and sexual drive can be affected by neurotransmitter monoamines such as serotonin, dopamine, adrenaline, noradrenaline and histamine. Evidence also showed that impulsivity, violence, and aggression are connected to serotonin deficiency (4). For cognitive abilities among patients with 48, XXYY, their measures of verbal IQ are lower than in their measures of performance IQ. Their verbal skills are relatively stronger compared to the visuospatial skills (5). Testosterone-based hormonal replacement therapy (HRT) has been proposed to improve the cognitive and behavioral profile including aggression (5). However, recent review (5) has suggested the need of further studies to establish the real impact of HRT on improving the cognitive and behavioral profile of these patients.

There is very little existing literature about psychiatric disorders among patients with 48,XXYY syndrome. There was a case report on a 28-year-old patient with underlying 48,XXYY syndrome who had been diagnosed with bipolar disorder, oppositional defiant disorder, learning disability, and mild intellectual disability. He was on a maintenance dose of lithium, which was subsequently discontinued due to an episode of renal failure (6). The authors of this case report highlighted the important comparisons between the phenotypes of classic 47,XXY karyotype and 48,XXYY syndrome. They also emphasized the importance of screening for ADHD, autism spectrum disorders, mood disorders, and other mental health problems.

The current case report focuses on a patient diagnosed with 48,XXYY syndrome who had his psychiatric care newly transitioned to an adult psychiatric clinic. It highlights the occurrence of aggression and disinhibited behavior, which were thought due to intellectual disability, ADHD, and 48,XXYY syndrome. The patient’s family had to endure difficulties in caring for the patient. However, when the presenting symptoms were accurately identified and the additional diagnosis of bipolar disorder was carefully formulated, the symptoms were remitted after appropriate treatment was started.

## 2. Case description

### 2.1. Case presentation

A 7-month-old floppy baby was initially investigated and a CT brain revealed cerebral atrophy. He was born via spontaneous vertex delivery and antenatally was uneventful. Based on the subsequent genetic study at 11 months, he was diagnosed with 48,XXYY syndrome. He started to walk at 2 years old and talk at 4 years old. Academically, he attended a special class which focus on skills training since he was 7 years old. He had frequent tantrums since he was 7 years old and started to display impulsive acts 1 year later. He was diagnosed with attention deficit hyperactive disorder (ADHD) and intellectual disability at 9 years old by a child psychiatrist. He started to smoke when he was 13 years old. He became impulsive, easily irritable, physically aggressive, and often threw a tantrum. He would be uncontrollably agitated if his needs were not approved, such as when he demanded money, cigarettes, and going out of the house. In view of his worsening symptoms, he was started on methylphenidate and referred to a clinical psychologist for his behavioral issues. However, these interventions were stopped because of minimal improvement. At 16 years old, he was started on sodium valproate to control his aggression and later on, haloperidol and risperidone were added.

He came to our attention at 19 years old for the continuation of treatment. He became talkative, spending more than usual, having a reduced need for sleep, and being easily irritable. Clinical examination revealed a tall young man with a height of 1.80 m and a weight of 106 kg (BMI = 32.71 kg/m^2^). His blood pressure was 143/75 mmHg, with a heart rate of 92 bpm. He had a long face with bitemporal narrowing, a prominent brow and chin. Despite being on triple medications (tablet sodium valproate 400 mg on morning and 600 mg at night, tablet haloperidol 2.5 mg at night and tablet risperidone 2 mg twice a day), he was reported to still be aggressive. However, his symptoms occurred intermittently, still manageable and did not warrant a hospital admission.

His condition remained stable on treatment until 2 years later, and his impulsivity significantly worsened. He intermittently demanded the purchase of expensive items. When his demands were not met, he would punch his father and brother and had to be physically restrained. There was also an increase in sexual drive manifested by his attempts to touch his female teacher inappropriately. These symptoms were episodic. Consequently, he was admitted to the psychiatric ward. It was observed that prior to these escalations of his symptoms, the patient experienced changes in his circadian rhythm because of disrupted sleep as he traveled from Malaysia to the United States of America for a trip. When he reached the USA, his symptoms emerged and worsened after he returned to Malaysia.

**FIGURE 1:**
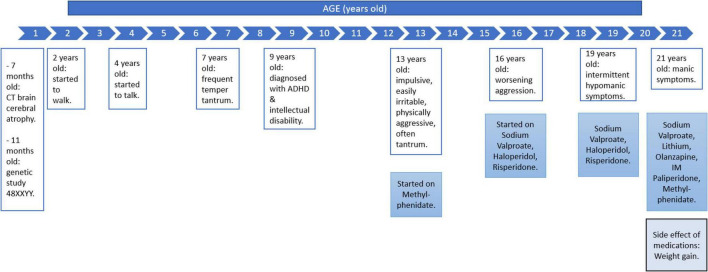
Timeline of the patient’s progress and medications.

In the ward, he appeared hostile yet was talkative, with coherent and relevant speech. He also had a flight of ideas with an expansive mood. Other system examinations were unremarkable. Further assessment revealed that he was experiencing other manic symptoms such as reduced need for sleep, high energy, and having many plans. He had no symptoms suggestive of autism spectrum disorder, conduct disorder, or personality disorder. He also had no history of trauma and substance abuse. He had a strong family history of bipolar mood disorder, whereby his maternal grandmother and auntie were diagnosed with the condition. His mother identified his behavior as similar to his grandmother (Figure 1).

### 2.2. Investigations

Blood investigations showed he had a deranged fasting lipid profile (total cholesterol was 5.66 mmol/L, triglyceride was 3.10 mmol/L, HDL was 0.92 mmol/L) and normal blood sugar level (HbA1c was 5.3% and fasting blood sugar of 4.88 mmol/L). CT scan of the brain revealed no focal enhancing brain parenchymal mass or other abnormalities (Table 1).

**TABLE 1:** Summary of investigations.

	19/9/2019	2/11/2019	3/12/2019	14/7/2020	10/9/2020	8/10/2020	20/11/2020	11/1/2021	21/4/2021	6/10/2021	19/5/2022	6/7/2022
Fasting blood sugar (mmol/L)	4.56					5.15				4.8		
HbA1c (%)								5.3				
Liver function test (ALP, ALT: U/L) (Bilirubin: umol/L)	ALP 76, ALT 14, Bilirubin 8.8		ALP 71, ALT 16, Bilirubin 6.4	ALP 67, ALT 16, Bilirubin 6.1	ALP 57, ALT 21, Bilirubin 5.4				ALP 71, ALT 30, Bilirubin 9.9		ALP 81, ALT 63, Bilirubin 8.4	ALP 73, ALT 25, Bilirubin 10.5
Lipid profile (mmol/L)	Cholesterol 5.93, HDL 1.01, LDL 4.11, TG 1.79	Cholesterol 5.00, HDL 0.76, LDL 3.68, TG 1.24				Cholesterol 5.17, HDL 1.10, LDL 3.17, TG 1.99		Cholesterol 5.66, HDL 0.92, LDL 3.33, TG 3.10	Cholesterol 4.35, HDL 0.85, LDL 2.20, TG 2.86		Cholesterol 5.94, HDL 1.16, LDL 3.60, TG 2.60	
Renal profile (Urea mmol/L, Creatinine umol/L, Na mmol/L, K mmol/L)	Urea 3.4, Creatinine 72.7	Urea 4.0, Creatinine 61.5		Urea 3.1, Creatinine 61.8, Na 139, K 4.3	Urea 3.9, Creatinine 69.7, Na 140, K 4.5			Urea 2.2, Creatinine 65.9, Na 139, K 4.6	Urea 2.8, Creatinine 71.8, Na 139, K 4.3	Urea 1.8, Creatinine 66.5, Na 140, K 4.6	Urea 2.8, Creatinine 71.8, Na 139, K 4.3	Urea 1.3, Creatinine 64.7, Na 138, K 4.1
Thyroid function test (FT4 pmol/L, TSH uIU/mL)	FT4 12.15, TSH 2.53			FT4 10.64, TSH 3.45						FT4 10.27, TSH 4.84		
Valproate level (umol/L)			476.8									

### 2.3. Diagnosis

At this point, we considered the possibility of comorbid bipolar disorder in this patient. It can be difficult to differentiate between behavioral problems with impulsivity that were due to intellectual disability, ADHD, or bipolar disorder. We argue that bipolar disorder should be considered based on: First, mood disorders were common among individuals with 48,XXYY syndrome, and were reported to be present in about a quarter of patients in a large case series (7). Second, as this patient was diagnosed with ADHD, it has also been reported that children with ADHD have a higher risk of developing bipolar mood disorder later in life due to shared genetic factors (8). In a recent meta-analysis of 71 studies with 646,766 participants from 18 countries, it was shown that 7.95% (one in 13) adults with ADHD were also diagnosed with bipolar disorder and 17.11% (one in six) adults with bipolar disorder had ADHD (9). Third, there is a strong genetic loading from two of the patient’s second-degree relatives from the maternal side. For twin studies, shared genes could explain the shared familial risk with around 70–90% heritability (10). The genetic predisposition can be explained by the evidence of a 10–15% risk of mood disorder among first-degree relatives of people with bipolar disorder demonstrated by family studies (10). We further postulated that the precipitating factor for the manifestation of the bipolar disorder diagnosis was the patient’s travel to the USA. It is known that bipolar episodes in susceptible people can be induced by circadian disruption in the form of jet lag especially those who fly across multiple time zones (11).

There is an overlap in diagnostic criteria, and the way out of the woods is to focus on treatment options and maintain vigilance for being inaccurate with one’s first diagnosis. For this patient, the presentations are suggestive of bipolarity instead of ADHD as the symptoms were episodic with the presence of the decreased need for sleep, increased rate of speech, and hypersexuality.

It needs to be acknowledged that the overall difficulty in managing challenging behavior in this patient was most likely multifactorial. The patient started to display impulsive behavior since he was 14 years old. When he was 19 years old, his impulsivity had worsened apart from sexual disinhibition and spending spree. These symptoms were initially attributed to ADHD and intellectual disability. Indeed, there are known factors correlated with challenging behavior in an adult with intellectual disabilities, including psychosocial vulnerabilities such as negative life events, impoverished social networks, lack of communication skills, lack of meaningful activities, and psychiatry or mood problems. Other factors are biological vulnerability factors include health, sensory, and genetic factors (12). Hence, methylphenidate was started and sodium valproate was increased, however, it was inadequate to control his aggression and manic symptoms, which led to the hospital admission. Considering these circumstances, the patient’s recent presentations were better explained by the additional diagnosis of the bipolar disorder instead of intellectual disability or ADHD alone. In other words, bipolar disorder may amplify behavioral problems due to intellectual disability and impulsivity due to ADHD.

### 2.4. Therapeutic interventions

The treatment plan for this patient was achieved by shared decision-making between the treating doctor, the patient, and his parents. The patient was started on tablet lithium 300 mg twice a day and intramuscular paliperidone 150 mg monthly during the admission while tablet olanzapine 10 mg daily and sodium valproate 600 mg in morning, 800 mg at night were continued. Lithium was added for the gold standard first-line treatment of bipolar disorder to control his mood symptom in manic and also maintenance phase (13). Paliperidone also has been listed in the treatment guideline for manic episode in bipolar disorder and intramuscular preparation helped to ensure compliance (13). He was discharged after 8 days of in-patient treatment (Figure 1).

### 2.5. Outcome and follow up

During his first follow-up, the manic symptoms had improved after lithium was initiated and optimized. There was still some spending spree. He used to order online food delivery, although most of the time he had no money to pay. With the improvement he achieved, he could embark on behavioral therapy. However, he was still in the early assessment phase and his progress in the therapy was suspended because of the lockdown measures imposed since the COVID-19 pandemic.

After optimization of his mood stabilizers, the patient was started on methylphenidate, and he could do his tasks at school better. His latest medications were a tablet lithium 300 mg in morning and 600 mg at night, sodium valproate 1,000 mg twice at night, intramuscular injection of paliperidone 150 mg monthly, olanzapine 5 mg twice a day, and methylphenidate extended release 36 mg daily. His latest lithium level was 0.99 mmol/L. In addition, he had been referred to a dietitian and a family physician for his metabolic conditions and his sugar cravings.

During the patient’s most recent outpatient follow-up, his condition was reported as improved as he would ask for permission from his parents before making purchases, and his parents could negotiate with him to limit the items which he intended to buy. Because of his improvement, the patient was also referred to a developmental psychologist for behavioral therapy. The long-term plan for this patient was to empower him to be independent of a personal caregiver, while a multidisciplinary team meeting between the patient’s family, his caregivers, psychiatrist, developmental psychologist, dietitian, and family medicine specialist was to be arranged in the near term.

## 3. Discussion

48,XXYY syndrome is considered a variant of 47,XXY Klinefelter syndrome due to a shared endocrinologic and physical phenotype. It was first described in 1964 and is associated with additional medical problems and more apparent psychological and neurodevelopmental features (5). The most common physiologic findings associated with 48,XXYY syndrome include an eunuchoid body habitus, small testes, azoospermia, gynecomastia, increased height, testicular atrophy following puberty, and increased follicular stimulating hormone (1). In addition, individuals with 48,XXYY syndrome were found to have significant developmental delays, problems with adaptive functions, and language-based cognitive deficits. Neurodevelopmental disorders are also common with the rates of ADHD reported at 72.2%, autism spectrum disorder at 18.9%, mood disorder at 26.3%, and tic disorders at 18.9%, with 55.9% of them on psychopharmacologic medications (5). Intellectual disturbances and behavioral issues among these patients are attributed to their sex-chromosomal abnormalities (5).

This case report highlighted diagnosing and treating bipolar disorder in a patient with 48,XXYY syndrome. His initial psychiatric presentation was aggressive behavior likely due to underlying intellectual disability and impulsivity associated with ADHD. Subsequently, these symptoms worsened, and manic-like symptoms became more pronounced, leading to his hospital admission.

In hindsight, this diagnosis of bipolar disorder might have been important in determining the patient’s clinical outcome. He experienced significant improvement in his manic symptoms, aggression, and disinhibition once he was started on lithium. Lithium is efficacious in reducing manic relapses and reducing the risk of relapse (14). In many individuals with 48,XXYY with specific psychiatric symptoms, including ADHD and mood disorders, psychiatric medications can be highly effective (15). Apart from the significant role of lithium in treating bipolar disorder, there was evidence of the effect of lithium in reducing aggressive behaviors in patient with intellectual disability and it was usually well-tolerated (16).

Another concern related to this patient is the risk of developing metabolic syndrome, as type 2 diabetes mellitus and obesity are common among 48,XXYY syndrome patients, although the mechanism is unknown (12). The International Diabetes Federation (IDF) stated that criteria for a patient to be diagnosed with metabolic syndrome include: the person must have central obesity plus any two of the following conditions: raised triglyceride, reduced HDL-cholesterol, raised blood pressure, and increased fasting plasma glucose (17). Metabolic syndrome prevalence has been found to be higher among patients with bipolar disorder (18). In a study that involved 972 younger bipolar and unipolar depressed patients in a current depressive episode, a higher metabolic syndrome prevalence was observed in both groups compared to the population controls (19). The patients had higher body mass index (BMI), higher glucose levels, total cholesterol, low-density-lipoprotein cholesterol (LDL), and lower high-density-lipoprotein cholesterol (HDL) but did not differ in blood pressure readings compared to the healthy controls. At the same time, the weight-gain promoting psychotropic agents for bipolar mood disorder also increases the risk for metabolic syndrome (20). Because of the additive risk, practitioners should weight the choice of medication dosage. Due to the risk of metabolic syndrome, which had been discussed earlier, the medications prescribed to him, including mood stabilizer and antipsychotics, need to be maintained at the minimum effective doses in order to find the balance between their risks and benefits.

The management approach for 48,XXYY patients should also include a multidisciplinary team approach (6), which may include geneticist, endocrinologist, family physician, psychiatrist, clinical psychologist as well as allied health professionals such as nutritionists, physiotherapist, occupational therapist, and psychologist. As in this case, he was referred to a family physician for the overall monitoring and treatment of his metabolic conditions, and risks of developing deep vein thrombosis (DVT) and non-Hodgkin lymphoma (7). A systematic review revealed that there was 18% prevalence of smoking among patients with intellectual disability (21). It is important to help this patient quit smoking as study has shown that 18% of 48,XXYY syndrome patients had deep venous thrombosis (7) and smoking would increase his risk further. The fact that the patient stopped smoking after his hospitalization had likely decreased the risk of DVT. On top of that, neuropsychological report deems needed to describe his neuropsychological profile, his strength and weakness as well as to be able to help the clinician in designing effective intervention. The report is also important in aiding the parents to equip their skill to handle the patient’s behavioral and emotional disturbance. However, neuropsychological assessment was not performed during writing of this report because the patient has mark generalized cognitive and learning deficit where he has been placed in special school.

Managing patients with rare diseases presenting with psychiatric conditions can be daunting for a psychiatrist in view of the absence of clear guidelines. In reporting this case, we hope to highlight the need for the early detection and prompt treatment of mood symptoms in patients with 48,XXYY syndrome by psychiatrists, family physicians, and other health professionals involved in their care. A multidisciplinary team-approach is probably the most ideal in managing the multiple medical, psychological, and social needs of patients with 48,XXYY syndrome. This case report was reported from the angle of psychiatry with collaboration with other discipline. This can be a guide for other psychiatrists. In the same time, this case report is discussing the overlapping issues of psychiatric conditions or other issued related with 48, XXYY individuals. On top of that, this case report is also highlighting the awareness among psychiatrist on the importance of monitoring for metabolic disease, not only because of the medications, but the condition itself predispose them to the metabolic disease.

## 4. Patient’s family perspective

### 4.1. Patient’s parents’ perspective

“At about age 13, our son displayed more disturbing symptoms of behavioral issues. He was becoming more aggressive and sometimes hostile when he got upset. He had difficulty playing for extended periods with peers due to his many developmental delays, thus making it challenging for him to interact with peers his age. Starting from about age 16, he also showed some sexual inclination which led to some incidents of inappropriate attempted sexual advances at his mother and once, a teacher at school. His manic episodes continue, with him talking excessively, showing loud behavior and having uncontrollable urge to splurge on purchases of anything that he fancies. Managing him on weekends and long school holidays, though is far more challenging as his manic tendencies meant that he was highly energetic and restless when there are no structured tasks. Having some semblance of a routine, i.e., bringing him along for weekly marketing and grocery shopping helped but managing him the rest of the day was often challenging to us. He liked being outdoors and always wanted to go out when family members needed some time to wind down and rest after a hectic workweek.”

## Data availability statement

The original contributions presented in this study are included in this article/Supplementary material, further inquiries can be directed to the corresponding author.

## Ethics statement

Written informed consent was obtained from the individual for the publication of any potentially identifiable data included in this article.

## Author contributions

NR, TM, NM and LW observed and treated the patient. All authors wrote the manuscript and approved the final work.
